# Spectral dependence of THz emission from InN and InGaN layers

**DOI:** 10.1038/s41598-019-43642-4

**Published:** 2019-05-08

**Authors:** Ričardas Norkus, Ramūnas Aleksiejūnas, Arūnas Kadys, Marek Kolenda, Gintautas Tamulaitis, Arūnas Krotkus

**Affiliations:** 1 Center for Physical Sciences and Technology, Saulėtekis av. 3, LT-10257 Vilnius, Lithuania; 2 Institute of Photonics and Nanotechnology, Vilnius University, Saulėtekis av. 3, LT-10257 Vilnius, Lithuania

**Keywords:** Semiconductors, Optical materials and structures

## Abstract

Spectral dependence of terahertz emission is a sensitive tool to analyze the structure of conduction band of semiconductors. In this work, we investigate the excitation spectra of THz pulses emitted from MOCVD-grown InN and InGaN epitaxial layers with indium content of 16%, 68%, and 80%. In InN and indium-rich InGaN layers we observe a gradual saturation of THz emission efficiency with increasing photon energy. This is in stark contrast to other III-V semiconductors where an abrupt drop of THz efficiency occurs at certain photon energy due to inter-valley electron scattering. From these results, we set a lower limit of the intervalley energy separation in the conduction band of InN as 2.4 eV. In terms of THz emission efficiency, the largest optical-to-THz energy conversion rate was obtained in 75 nm thick In_0.16_Ga_0.84_N layer, while lower THz emission efficiency was observed from InN and indium-rich InGaN layers due to the screening of built-in field by a high-density electron gas in these materials.

## Introduction

Excitation of semiconductor surfaces with femtosecond (fs) laser pulses is an important way of generating ultrafast electromagnetic transients with characteristic spectra reaching into the terahertz (THz) frequency range^1^. This effect is exploited for producing the THz emission sources^2^. The surface THz emission is caused by several physical processes and, thus, can be used as an experimental tool for investigating various semiconductor material parameters. In particular, THz emission efficiency is very sensitive to electron mobility allowing for investigations of subsidiary valleys in the conduction band^3–5^ and offset energy in heterostructures^6^ from the dependence of THz emission efficiency on optical excitation wavelength.

Indium nitride is a narrow bandgap (~0.7 eV) semiconductor with high intrinsic electron mobility (3500 cm^2^/V·s at room temperature has been measured in thick MBE-grown layer^7^). As such, InN is considered for many applications, including infrared photonic devices^8–10^, solar cells^9,11^, and THz emitters^12–14^. Unfortunately, its potential has not been realized up to now as it suffers from extremely high unintentional n-type doping, usually in the range of 10^19^ cm^−3^. In highly doped InN structures, the electron mobility is reduced tenfold or even more due to carrier scattering by ionized impurities^15^. The THz emission efficiency is also diminished by free electrons that screen the built-in electrical field; the efficiency can be in part restored by doping with magnesium, though^14,16–18^. The unintentional doping is extremally difficult to control in InN grown by metalorganic chemical vapor deposition (MOCVD). Several technological approaches have been tested to improve the quality of MOCVD layers, including post-growth annealing^15^, growth temperature ramping, and interrupted growth^19^; however, the electron density remains in the mid-10^18^ cm^−3^ range even in the best MOCVD InN layers^15^.

Among other advantages, InN (like other III-nitrides) is reported to have high values of electron saturation velocity^20^, mainly due to the fact that subsidiary conduction band valleys are located at high energies. Recent analysis of wurtzite InN by W. Hadi *et al*. predicts two lowest subsidiary valleys at Γ_2_ and *L* − *M* points, with intervalley energy separation of 1.8 and 2.7 eV from the main minimum at Γ_1_, which is 0.7 eV above the valence band^21^. Large energy separation of higher energy minima makes it difficult to observe them experimentally, since usually the tunability range of laser excitation sources is limited. Several attempts were carried out to measure the position of the subsidiary valleys in InN by using the spectrally-dependent THz emission technique, but the highest excitation photon energy did not exceed 1.7 eV, obscuring the observation of intervalley scattering effects^5,16,22^.

In this work, we investigate the dependence of THz surface emission efficiency on excitation wavelength in InN and InGaN layers, using the pump photon energy up to 3.0 eV. To simultaneously reveal the impact of free electrons to THz emission, we compare InN, indium-rich InGaN, and gallium-rich InGaN layers on c-sapphire. We demonstrate and discuss the different nature of spectral dependencies of THz emission in InN and InGaN layers. In InN, the gradual saturation of THz emission and its subsequent decrease with photon energy is likely to be caused by increase of electron effective mass in non-parabolic conduction band. An abrupt drop in THz emission in indium-poor layer is attributed to electron transfer to GaN substrate instead and used to estimate the InGaN/GaN offset.

## Materials and Methods

Four epilayers are studied in this paper: InN layer, two In-rich layers In_0.68_Ga_0.32_N and In_0.80_Ga_0.20_N, and one Ga-rich layer In_0.16_Ga_0.84_N. All samples where grown in shower-head type MOCVD reactor (AIXTRON) on (0001) GaN/sapphire templates. The thickness of GaN buffer layer was 5 μm for InN layer and 3.5 μm for InGaN layers. In_0.16_Ga_0.84_N samples were grown using continuous flow of precursors, while the InN and In-rich samples were grown using the interrupted growth method. The interrupted growth was used in order to decrease the unintentional n-type doping in InN layers and to prevent the indium segregation in indium-rich InGaN layers; for the detailed description of the growth procedures please refer to refs^19,23^. The thickness of layers was 75 nm for In_0.16_Ga_0.84_N, 200 nm for In_0.68_Ga_0.32_N and In_0.80_Ga_0.20_N, and 400 nm for InN. The main parameters of the studied structures are listed in the Table 1.

**Table 1:** Main parameters of the studied samples.

Epilayer	Layer thickness, nm	PL peak, nm	Electron Hall density, ×10^19^ cm^−3^	Electron Hall mobility, cm^2^/Vs
In_0.16_Ga_0.84_N	75	466	N/A	N/A
In_0.68_Ga_0.32_N	200	1127	~8.0	<100
In_0.8_Ga_0.2_N	200	1320	3.4	141
InN	400	1670	1.3	598

Surface THz emission experiments have been performed in a quasi-reflection geometry with samples illuminated at 45° to their surface normal. The experimental setup is based on an amplified ytterbium-doped potassium gadolinium tungstate (Yb:KGW) laser system (PHAROS, Light Conversion Ltd.) operating at 1030 nm with pulse duration of 160 fs and repetition rate of 200 kHz. Average power of 6 W from the laser is directed into a cavity-tuned optical parametric amplifier (OPA, ORPHEUS, Light Conversion Ltd.) that generates 140–180 fs duration pulses with a central wavelength tunable from 640 nm to 2600 nm. Shorter wavelengths of femtosecond optical pulses - up to 400 nm - were reached by generating the second harmonic in a BBO (Beta Barium Borate) crystal. In the THz-TDS arrangement, the samples were excited by the OPA output beam, while the sample-emitted THz pulses were detected by the GaAsBi photoconducting antenna (TeraVil Ltd.). The average power of the optical pulses incident on a sample was about 10 mW. The THz detector was illuminated by a small fraction of Yb:KGW laser beam (the average power of ~5 mW), which was delayed by different amount of time with respect to the optical beam exciting the sample. All experiments were performed at room temperature.

## Results and Discussion

**Figure 1:**
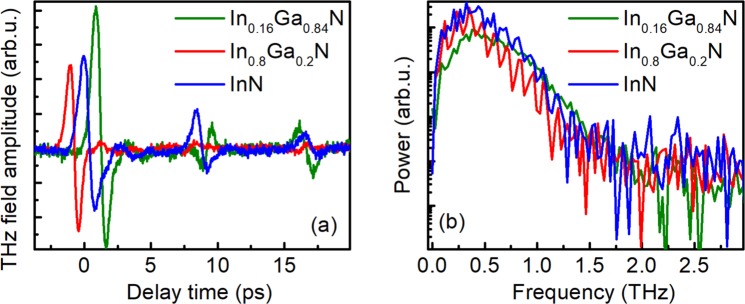
Temporal shape of THz pulse (**a**) and its Fourier spectrum (**b**) emitted from In_0.16_Ga_0.84_N (green lines), In_0.8_Ga_0.2_N (red lines), and InN (blue lines) samples excited by the 10 mW average power femtosecond optical pulses with wavelengths corresponding to THz excitation spectra maxima.

Figure 1 presents the typical shapes and their Fourier spectra of THz pulses emitted by three nitride samples. These pulses were measured at optical wavelengths *λ* corresponding to the maxima of THz excitation spectra of these samples: λ = 520 nm for InN, λ = 360 nm for In_0.8_Ga_0.2_N, and λ = 380 nm for In_0.16_Ga_0.84_. All measurements were performed at an average optical beam power of 10 mW; the curves in Fig. 1 are normalized to a constant photon number per optical pulse.

As measured, the amplitudes of THz pulses emitted from all samples were of the same order of magnitude, but the efficiency of optical-to-THz pulse conversion was the largest in In_0.16_Ga_0.84_N sample. The bandwidths of the Fourier spectra reached ~2 THz and the signal-to-noise ratio was approximately equal to 30 dB. The spectra of both In-rich InGaN samples were modulated due to the influence of THz pulse reflections in the substrate. One has to point out that the signal-to-noise ratio obtained under the same conditions in a p-type InAs crystal (the hole density ~10^16^ cm^−3^) was of the order of 50 dB (not shown), and the amplitude of THz pulses emitted from the latter crystal was ~10 times larger than that obtained in the nitride samples.

**Figure 2:**
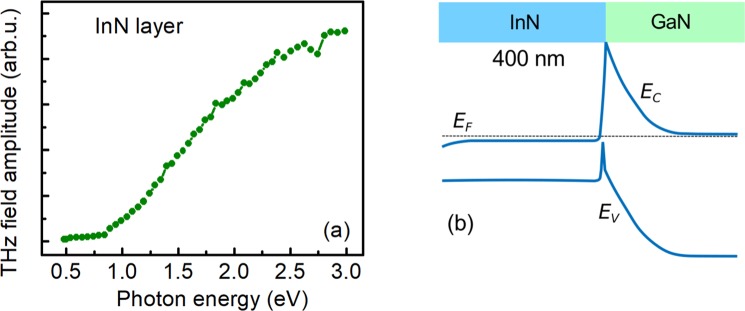
(**a**) The amplitude of THz field as a function of excitation photon energy in InN layer. (**b**) The modelled energy bands in the InN layer.

THz excitation spectrum of InN layer is shown in Fig. 2(a), while Fig. 2(b) presents the modelled energy band structure of this sample. The energy band structure was calculated using the one-dimensional Schrodinger-Poisson equation solver^24^. The onset of THz emission at the photon energies of ~1 eV is followed by a sublinear growth of THz field amplitude with increasing photon energy and its saturation at the end of the measurement range of ~3 eV. Because of the high doping level and short (~4 nm) Debye length in this sample the energy bands are flat and any built-in electrical fields are screened. Therefore, the most probable physical mechanism of surface THz emission is the photo-Dember effect – electron and hole spatial separation due to their different diffusion rates. This effect starts to manifest itself when the light absorption length becomes shorter than the width of the sample – this explains why the onset of THz emission occurs at photon energies exceeding the band gap (photoluminescence peak in this sample is at ~0.74 eV).

THz surface emission spectra measured in other group III-V bulk semiconductors^3,4,25^ have clear maxima at the photon energies *hv*_12_ corresponding to photoexcitation of electrons from the valence band to the subsidiary, higher effective mass conduction band valleys. By using the energy and momentum conservation laws, this photon energy can be used to calculate the energy separation *ε*_12_ between the main Γ valley and the subsidiary valleys in the non-parabolic, Kane type conduction band using the relation^25^:

1 $${\varepsilon }_{12}=\frac{-1-\frac{{m}_{{\rm{e}}}}{{m}_{{\rm{h}}}}+\sqrt{{(1+\frac{{m}_{{\rm{e}}}}{{m}_{{\rm{h}}}})}^{2}+4\frac{{m}_{{\rm{e}}}}{{m}_{{\rm{h}}}}\beta (h{\nu }_{12}-{E}_{{\rm{G}}})}}{2\frac{{m}_{{\rm{e}}}}{{m}_{{\rm{h}}}}\beta }-{\varepsilon }_{f}$$

here *m*_e_ and *m*_h_ are the electron and heavy hole effective masses, *E*_*G*_ is the energy band gap, *ε*_*f*_ = 73 meV is the intervalley phonon energy, and *β* = 1/*E*_*G*_– the nonparabolicity parameter.

We use Eq. (1) to investigate if the saturation of THz signal at 3 eV can be caused by electronic transitions to subsidiary conduction band valleys. It has to be noted that there is an uncertainty as to the values of InN hole effective mass, due to its strong anisotropy and dependence on strain. In this work, we use the calculated hole effective mass for the direction parallel to c plane (the direction of hole movement in our sample) *m*_h_ = 1.86*m*_0_; here *m*_0_ is the free electron mass^26^. The other parameters used for calculations were: *m*_e_ = 0.05*m*_0_, *β* = 1.43, *E*_G_ = 0.7^27^. Using Eq. (1) and the photon energy *hv*_12_ = 3 eV we find the lower limit of the intervalley separation energy in the conduction band as *ε*_12_ > 2.4 eV. The two closest subsidiary conduction band minima are calculated to be at points Γ_2_ (energy separation from Γ_1_ is *ε*_12_ = 1.78 eV) and at *L* − *M* (*ε*_12_ = 2.71 eV)^28^.

We attribute the sublinear growth and the saturation of THz signal to a gradual increase of electron mass with the energy, due to strong conduction band non-parabolicity. As it has been shown in ref.^29^, the electron energy dispersion in InN can be described by the two band Kane’s law^30^; therefore, electron effective mass will depend on the energy *ε* as:

2 $${m}_{e}(\varepsilon )={m}_{e}(0)[1+2\beta \varepsilon ]$$

As it follows from this equation, effective mass of an electron with the energy of 2.4 eV is nearly 7 times larger than its value at the conduction band edge. Therefore, a monotonous increase of electron mass can lead to the saturation of a photoexcited electron mobility and strength of a dynamic dipole, caused by the electron and hole separation, rather than their abrupt decrease.

**Figure 3:**
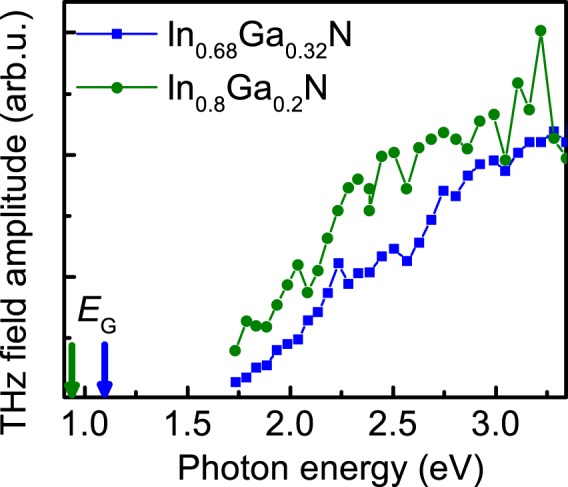
The amplitude of THz field as a function of excitation photon energy in indium-rich In_x_Ga_1−x_N layers with x = 0.68 (blue symbols) and x = 0.8 (green symbols).

THz excitation spectra of two In-rich InGaN samples are presented in Fig. 3. Because the width of nitride layers in these samples is only 200 nm, difference between the energy at which THz emission sets on and the energy bandgap is larger than that in 400 nm thick InN layer. In-rich InGaN samples also have high n-type doping level comparable to that in InN layer; therefore, they are characterized by the similar flat-band structure shown in Fig. 2(b). The excitation dependence of THz emission in indium-rich InGaN layers strongly resembles that in InN layer; one can assume that the dominant process of THz emission in these layers is photo-Dember. The saturation of THz emission with increasing pump photon energy is still present, but this effect gradually vanishes as indium content is decreased, most likely due to the increasing energy gap.

**Figure 4:**
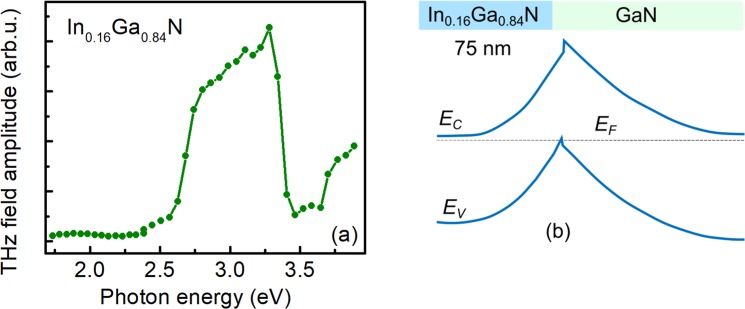
THz emission intensity dependence on excitation photon energy (**a**) and the modelled energy bands (**b**) in In_0.16_Ga_0.84_N layer.

THz excitation spectrum of the InGaN sample with In content of 16% is different from all the spectra described above. THz emission starts at photon energies close to the bandgap of this layer, which was estimated to be 2.78 eV, assuming the strained layers^31^. This fact evidences that the origin of THz emission is photocurrent surge in the built-in electric field rather than the photo-Dember effect. Thin InGaN layer in this sample prevented the Hall measurements, however, one can assume that the residual doping level in In_0.16_Ga_0.84_N sample is much lower than in In-rich samples, of the typical order of (1–3)×10^17^ cm^−3^. The potential profile calculated by assuming this level of doping is shown on Fig. 4(b) allows to explain all main features of the spectrum presented on Fig. 4(a).

A steep rise of the measured peak-to-peak amplitude of THz transients corresponds to the energy bandgap of InGaN as determined from the photoluminescence measurement. A similarly steep decrease is observed when some of the photoelectrons excited in InGaN layer become transferred to GaN, where they move in the opposite direction than the electrons left in the InGaN. This decrease begins at the photon energies of ~0.16 eV lower than the bandgap of GaN, which corresponds to the valence band offset at the heterointerface between In_0.16_Ga_0.84_N and GaN^32^. A final, rising part of the THz excitation spectrum is caused by the acceleration of the electrons excited in GaN.

In summary, THz pulse emission from InN and InGaN epitaxial layers was measured over a wide wavelength range of femtosecond optical pulses used for their photoexcitation. In InN and In-rich InGaN layers, the amplitude of THz pulses was gradually saturating at high photon energies instead of rapidly decreasing, as it happens in the majority of other group III-V semiconductors due to inter-valley electron scattering. This observation allows us to set the lower limit of the intervalley energy separation in the conduction band of InN as 2.4 eV.

The efficiency of THz generation in investigated InN and In-rich InGaN layers was seriously restricted by their high doping level and dynamical screening of the appearing electrical dipoles by the equilibrium electrons. More efficient performance of these materials as THz emitters could be possible if layers with compensated residual impurities would be used. Such a structure containing only a 75 nm thick In_0.16_Ga_0.84_N layer grown on GaN template was emitting THz pulses more efficiently than 5 times thicker InN layer. This efficiency can be further improved by a proper use of the piezoelectric fields arising at the interfaces between different nitride layers.

## Data Availability

All data analyzed during this study are included in this published article.
